# Conceptualizing soil fauna effects on labile and stabilized soil organic matter

**DOI:** 10.1038/s41467-024-49240-x

**Published:** 2024-06-17

**Authors:** Gerrit Angst, Anton Potapov, François-Xavier Joly, Šárka Angst, Jan Frouz, Pierre Ganault, Nico Eisenhauer

**Affiliations:** 1 German Centre for Integrative Biodiversity Research (iDiv) Halle-Jena-Leipzig, Puschstraße 4, 04103 Leipzig, Germany; 2 Institute of Biology, Leipzig University, Leipzig, Germany; 3 Biology Centre of the Czech Academy of Sciences, Institute of Soil Biology & Biogeochemistry, Na Sádkách 7, 37005 České Budějovice, Czech Republic; 4 Institute for Environmental Studies, Charles University, Benátská 2, Praha 2, Prague, Czech Republic; 5 Senckenberg Museum für Naturkunde Görlitz, Postfach 300 154, 02806 Görlitz, Germany; 6 Eco&Sols, Univ Montpellier, CIRAD, INRAE, Institut Agro, IRD, Montpellier, France; 7 Laboratoire ECODIV USC INRAE 1499, Université de Rouen Normandie, FR CNRS 3730 SCALE, Rouen, France

**Keywords:** Biogeochemistry, Carbon cycle, Element cycles, Carbon cycle

## Abstract

Fauna is highly abundant and diverse in soils worldwide, but surprisingly little is known about how it affects soil organic matter stabilization. Here, we review how the ecological strategies of a multitude of soil faunal taxa can affect the formation and persistence of labile (particulate organic matter, POM) and stabilized soil organic matter (mineral-associated organic matter, MAOM). We propose three major mechanisms - transformation, translocation, and grazing on microorganisms - by which soil fauna alters factors deemed essential in the formation of POM and MAOM, including the quantity and decomposability of organic matter, soil mineralogy, and the abundance, location, and composition of the microbial community. Determining the relevance of these mechanisms to POM and MAOM formation in cross-disciplinary studies that cover individual taxa and more complex faunal communities, and employ physical fractionation, isotopic, and microbiological approaches is essential to advance concepts, models, and policies focused on soil organic matter and effectively manage soils as carbon sinks, nutrient stores, and providers of food.

## Abstract (web summary)

In their review, Angst et al. conceptualize how the ecological strategies of a multitude of soil faunal taxa can influence the formation of particulate and mineral-associated organic matter. The authors highlight research gaps and ways forward.

## Introduction

Soil fauna is highly abundant, diverse, and active in soils worldwide, even in the most extreme environments, such as Antarctica or deserts^1–5^. Soil fauna affects various soil biophysicochemical properties, including microbial diversity, soil structure/texture, and soil nutrients, via ingesting and transforming organic matter into more or less decomposable forms, mixing this organic matter with mineral soil, and grazing on microorganisms^6–10^. These processes are essential in driving biogeochemical cycles and may substantially affect soil organic matter (SOM) dynamics^11–16^. However, despite this recognized relevance of soil fauna to soil processes, knowledge of its involvement in the formation and stabilization of SOM is very scarce, except for that of earthworms^17–22^. This key knowledge gap hampers our understanding and modeling of global biogeochemical cycles and thus effective management of soils.

Current paradigms consider the formation of stabilized SOM to be regulated by the efficiency with which microorganisms transform plant litter (leaf and root) and root exudates into microbial biomass^23–25^, which is strongly affected by litter/exudate quality^26^. When microbial biomass eventually turns into necromass, it readily interacts with mineral surfaces and thus accumulates as mineral-associated organic matter (MAOM; “microbial pathway” of SOM formation), which can persist for centuries to millennia^27^ (see also Kleber et al.^28^). This stabilized SOM pool can also form via direct sorption of dissolved organic matter on reactive mineral surfaces, which requires little or no microbial pre-processing of organic matter (“direct sorption pathway” of SOM formation)^29,30^. Notably, soil fauna might substantially affect both of these SOM formation pathways by (1) altering the quantity, quality, and location of organic matter in the soil via bioturbation and transformation processes, (2) altering microbial biomass and community composition via active and/or indirect grazing on bacteria and fungi, (3) generating dissolved organic matter via processing of litter and other organic matter, and (4) influencing soil texture (and mineralogy)^6,31–33^. All of these processes also have the potential to affect the share of comparatively labile pools in soils, such as particulate organic matter (POM). This pool is composed of partly decomposed plant fragments that have, if not occluded within aggregate structures, comparatively short residence time in soils (<15 years)^34–36^.

Particulate OM is directly linked to the formation of MAOM, in which effective decomposition of bioavailable POM can boost MAOM formation via the microbial pathway, while “recalcitrant” POM can hamper the formation of MAOM but foster relative accumulation of POM^37^. The proportion of labile (POM) and stabilized pools of SOM (MAOM) determines how this SOM, and carbon (C) within, responds to altered environmental conditions, such as induced by land use, climate change^38^, or management focused on increasing or maintaining C storage^37^, nutrient stores, or crop yields^39^.

**Fig. 1:**
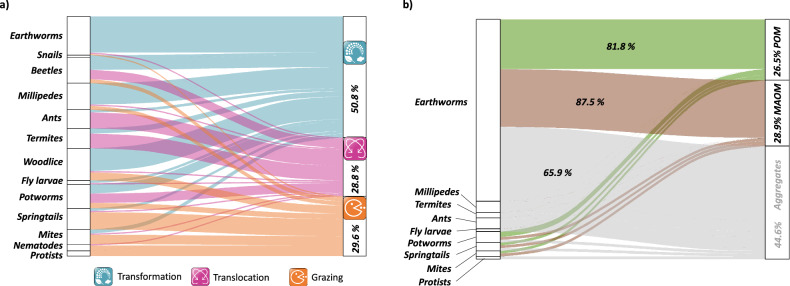
Overview of studies on soil fauna. **a** Studies by process (translocation, transformation, grazing; *n* = 180) and **b** investigated fraction (POM, MAOM, aggregates; *n* = 87) based on our literature search; % values in **a** indicate the proportion of studies (research articles) dedicated to translocation, transformation, and/or grazing independent of taxon, respectively. Percentage values in **b** to the right indicate the proportion of studies that investigated POM, MAOM, and aggregates independent of taxon, respectively, while % values within the alluvial graph indicate the proportion of studies on earthworms relative to the whole number of studies. Aggregates are grayed out because they were not in the focus of our review (consisting of both POM and MAOM).

**Fig. 2:**
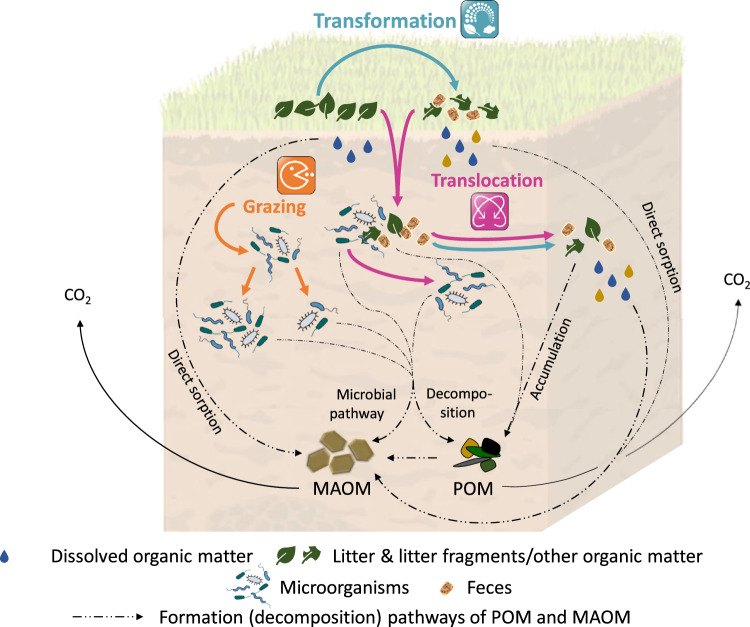
Soil fauna affects formation pathways of POM and MAOM via transformation, translocation, and grazing. Soil fauna transforms litter and other organic matter into more or less decomposable forms, such as feces and litter fragments. This organic matter is then translocated via bioturbation into the mineral soil, where it can be further transformed or translocated vertically or horizontally and eventually accumulate as POM (as “free” POM or occluded within aggregates). This POM can then be microbially processed and either be stabilized as MAOM or mineralized to CO_2_. Transformation of organic matter by soil fauna can also result in the release of dissolved organic matter from litter layers or, specifically, feces (both in the litter layer or mineral soil), which may have altered chemistry compared to the initial litter (indicated by different drop colors). This dissolved organic matter can directly sorb on mineral surfaces and thus form MAOM and/or desorb previously sorbed organic matter, which can then be mineralized to CO_2_. Grazing on and translocation of microbial communities by fauna may affect microbial physiological traits and community composition and abundance and thus have an influence on the microbial pathway of MAOM formation (and decomposition of POM).

**Fig. 3:**
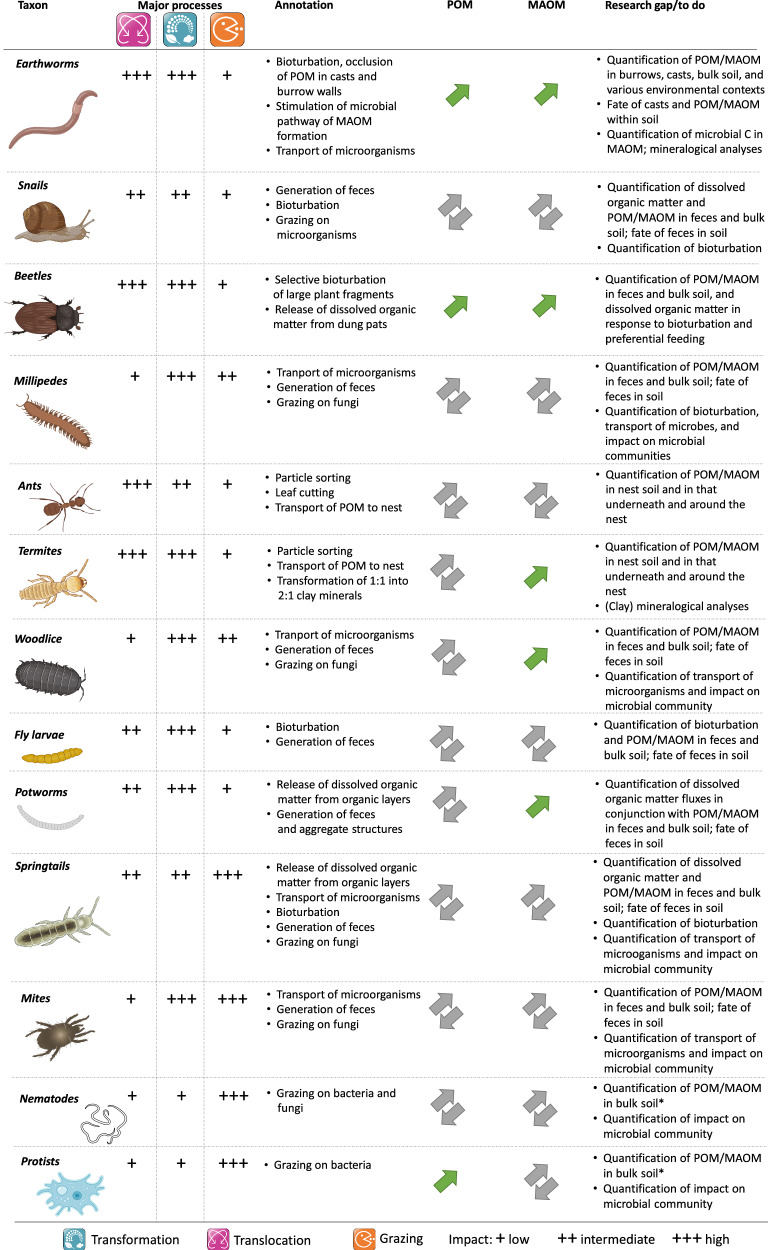
Potential impact (low, intermediate, high) of the main processes (transformation, translocation, grazing) by which faunal taxa can influence POM and MAOM, direction of the faunal effect (green arrows indicate positive, gray arrows neutral or unknown effects on POM or MAOM), and remaining research gaps. *Investigation of POM and MAOM in feces of nematodes and protists is likely unfeasible due to the small amounts of feces produced by these taxa relative to the amount necessary for physical fractionation. Some icons have been created using BioIcons.com (DBCLS; springtails and termites; CC-BY 4.0) and BioRender.com (earthworms, snails, beetles, ants, woodlice, protists). Icons created with BioRender.com are released under a Creative Commons Attribution-NonCommercial-NoDerivs 4.0 International license.

Despite strong indications that soil fauna may substantially affect the pathways of MAOM and POM formation, knowledge on these effects, except for earthworms, is virtually absent (Fig. 1). We argue that such knowledge is crucially needed to manage soils as C sinks and accurately predict SOM dynamics in the face of climate change. In this review, we summarize the state of the art of how macro-, meso-, and microfauna affect soil properties, such as SOM quantity and chemistry, microbial biomass and community composition, or soil texture. We categorize these effects into three main processes - transformation, translocation, and grazing - by which soil fauna can alter the share between and the formation of POM and MAOM (Fig. 2). We discuss how these processes can be altered by interactions among soil faunal taxa and environmental change and propose future research directions (Fig. 3).

We ultimately call for laboratory and field studies that focus on the direction and magnitude of transformation, translocation, and grazing effects of individual soil faunal taxa and whole communities on the formation and/or decomposition of POM and MAOM, and how environmental factors such as land use, climate, or soil type alter these processes. The results of such studies are envisioned to help refine current concepts on SOM formation and soil organic C-related models to be eventually implemented at the management and policy level.

## Literature review

We focused our systematic literature search on soil macro-, meso-, and microfaunal taxa with high abundance in soil and potential influence on POM and MAOM^40^, including ants (Formicidae), beetles (Carabidae), earthworms (Lumbricina/Crassiclitellata), fly larvae (Diptera), millipedes (Diplopoda), mites (mainly Mesostigmata/Prostigmata/Oribatida), nematodes (Nematoda), potworms (Enchytraeidae), protists (Protozoa), snails (Gastropoda), springtails (Collembola), termites (Isoptera), and woodlice (Isopoda). We used strings [“TITLE-ABS-KEY (//taxon AND soil AND fraction* OR MAOM OR POM OR soil organic matter OR SOM OR carbon OR nitrogen OR phosphorus OR lipid* OR lignin OR protein OR necromass OR aggregate OR “Organo-mineral association*“ OR “mineral-associated organic matter” OR NMR OR FTIR OR GCMS OR biomarker* OR “microbial biomass” OR “microbial carbon” OR fung* OR bacteria*)” and “((AB = (//taxa) AND AB = (soil) AND AB = (fraction*) AND AB = (MAOM OR POM OR soil organic matter OR SOM OR carbon OR nitrogen OR phosphorus OR lipids OR lignin OR protein OR necromass OR aggregate OR “organo-mineral association*“ OR “mineral-associated organic matter” OR NMR OR FTIR OR GCMS OR biomarker* OR “microbial biomass” OR “microbial carbon” OR fung* OR bacteria*)”] to scan the Scopus and Web of Science databases for suitable articles and complement our expertize on the topic. Although soil fauna may also influence the formation and share of POM and MAOM via effects on plant growth and resource allocation^41–43^, we did not include a detailed review of this topic because it is beyond the scope of this study. The initial number of articles returned was 1,794 and 1811 for the Scopus and Web of Science databases, respectively. After screening of titles and abstracts and excluding articles out of scope, we finally based our review on 180 core articles about the effects of one or more faunal taxa on one or more soil properties related to SOM formation (Table S1). We then read the whole articles and extracted information on the types of soil fauna and their major effects on the soil properties investigated.

Based on this review of literature and our expertise, we identified three major processes by which soil fauna may influence formation of POM and MAOM: (i) transformation, in which soil fauna alters soil mineralogy or transforms organic matter into more or less decomposable forms via ingestion, digestion, and egestion, (ii) translocation, in which soil fauna moves soil particles, organic matter, or microorganisms from one location to another, and (iii) grazing on microorganisms, in which soil fauna modifies soil microbial community composition, biomass, or activity via targeted or incidental grazing. Many soil faunal taxa perform more than one of these processes and are thus repeatedly referred to in the following sections (cf. Fig. 1).

## Transformation

### Feces/casts

An important process by which soil fauna can influence the chemistry and stability of SOM is the ingestion of mineral particles and/or litter/organic matter, partial digestion in the gut, and egestion of the altered material as feces (Fig. 2). Depending on the ingested material, taxon and environmental context, this process can result in more labile, recalcitrant, or stabilized organic matter in feces compared to the ingested material.

#### Earthworm casts

The most prominent and researched feces (commonly termed casts) are those of earthworms. Earthworms burrow through the soil and ingest SOM or litter and/or mineral particles and mix this material with mucus in their guts. The egested casts often turn into stabilized (micro-)aggregate structures upon ageing^44–49^, in which POM is occluded (i.e., protected from decomposition), and in which MAOM is more stable as compared to that outside of aggregates^50^. Casts can have a distinct molecular composition compared to uningested bulk soil^9,19,47,51–55^, with enhanced microbial diversity, richness, and activity, and altered community composition^56–58^, and are often enriched in microbial biomass^59–62^. This earthworm-induced stimulation of the soil microbial community has recently been conceptualized to boost the microbial pathway of MAOM formation^63^, as earthworms co-locate microorganisms, microbial energy sources, and mineral particles, and potentially alleviate nutrient limitations by the addition of mucus. This is supported by studies showing increased microbial C use efficiencies^64,65^, microbial necromass (which preferentially associates with minerals)^19,22^, and MAOM in casts^20,66^ as compared to non-ingested soil. The stabilizing effect of earthworms on both POM and MAOM is highly relevant to SOM dynamics, given the high earthworm abundance and biomass in many soils worldwide^2,63^. However, earthworm-induced stimulation of soil microbial activity and biomass can also lead to increased mineralization and emission of CO_2_ and/or N_2_O from soils^67–70^. The net effect of earthworms on the C balance of soils is not solved and may be strongly modulated by the respective environmental conditions (e.g., climate, soil properties, plant, and faunal communities), experimental design (length, water regime, SOM content), and earthworm ecological groups, species, and/or traits^71,72^. More, preferably long-term, field studies involving earthworms (and other soil fauna) and quantification of C fluxes and pools are clearly needed (Fig. 3). Moreover, research on earthworm casts typically compares cast properties to those of uningested bulk soil, thereby omitting the contribution of ingested litter (e.g., leaf or root litter) to cast properties. Future studies should thus compare cast properties with those expected from all ingested substrates (decomposing litter and soil) weighed by their ingestion rate, or at least with a control mixture of mineral soil and litter^19^.

#### Millipedes, woodlice, snails, fly larvae, springtails, mites, termites, and potworms

Apart from earthworms, many other faunal taxa may also substantially affect SOM dynamics via the production of feces. Because most other soil faunal taxa are more abundant in the litter layer and feed on decomposing dead leaves, studies typically compared feces properties to those of uningested litter. Generally, feces of taxa other than earthworms feature physicochemical characteristics that are favorable for microbial proliferation and leaching as compared to non-ingested litter. These include higher nitrogen or phosphorus contents, lower contents of recalcitrant compounds such as lignin and secondary metabolites, higher contents of dissolved organic C and nitrogen, and higher surface area and water-holding capacity. Such features have often been reported for feces of millipedes^73–79^, woodlice^6,80,81^, snails^6,80^, and partly, mites^82^. In contrast, some studies on woodlice and fly larvae reported feces properties rather unfavorable for microbial proliferation, such as higher contents of lignin or lower contents of nitrogen as compared to non-ingested litter^81,83–88^. These contrasted results, however, do not appear to be taxa-specific but depend on the age of feces^83,84^ and the quality of ingested litter. Indeed, Joly et al.^6^ reported that across diverse taxa (millipedes, woodlice, and snails), the positive conversion effect on feces quality was stronger for low- than for high-quality litter. The magnitude of the change in quality, however, was species-specific, indicating that generalizations across species and taxa remain difficult.

The changes in physicochemical characteristics following litter conversion into feces may have important consequences for the gradual transformation of litter into SOM. Several studies found that these changes were associated with generally higher mass loss rates during decomposition for feces compared to uningested litter^6,75,77^, with only few cases of slower decomposition for feces compared to uningested litter^84^. These decomposition rates may be related to the addition of gut microbiota involved in the degradation of organic matter^73,89^ and facilitated leaching of dissolved organic compounds^6^. This suggests that conversion of litter into feces, by facilitating microbial proliferation and/or leaching, may boost the production of microbial necromass and leachates and thus the stabilization of litter as MAOM via the microbial and/or direct sorption pathways. Alternatively, conversion into feces, being composed of a myriad of minute particles, may increase the accumulation of POM, specifically for those feces that decompose more slowly than the uningested litter. Feces may also form nuclei for aggregate formation, such as reported for springtails, millipedes, potworms^90–93^, or constitute microaggregates themselves, as reported for termites^94^, thus increasing the persistence of POM within. The conversion effect of litter into feces on MAOM formation and accumulation of POM may also depend on the location at which feces are produced. If feces are located in or transferred to the mineral soil, actively or passively (e.g., via ingestion and transport by earthworms), they may affect both POM and MAOM. Feces remaining on the soil surface, however, may affect MAOM mainly via dissolved organic matter but have likely little effect on POM in mineral soil layers.

Feces-related research on taxa other than earthworms has mainly been focused on the conversion of litter into feces in the absence of mineral soil^6^. While this provides valuable information on physical and chemical changes of litter upon ingestion, digestion, and egestion, this does not allow to track the ultimate fate of these feces, i.e., their mineralization to CO_2_ or transfer to and stabilization in mineral soil. On the other hand, earthworm-related research has focused more on SOM dynamics in mineral soil, with less emphasis on litter decomposition. We thus advocate for a systematic consideration of litter *and* mineral soil in studies on the feces of earthworms and other taxa to identify general effects across the diversity of faunal taxa on the formation of POM and MAOM (Fig. 3).

### Mineral composition

Mineral composition affects the reactive surface area available for the sorption of organic matter^95^. Few faunal taxa can directly change this composition and affect the capacity of soils to store organic matter in MAOM. Under controlled conditions, Jouquet et al.^7,96^ showed that termites were able to extract otherwise un-exchangeable potassium from illites likely via saliva and stimulation of microorganisms, thus forming expandable smectite layers, which have higher capacity to sorb organic matter^97^. This process may explain the enrichment of expandable clays and the higher cation exchange capacity observed in termite nests^98,99^ and potentially increase MAOM formation. Earthworm activity may also alter mineral weathering^100,101^, with yet unknown consequences for the sorption of organic matter. Studies on how certain faunal taxa such as termites and earthworms affect MAOM formation may thus want to involve mineralogical analyzes (e.g., via X-ray diffraction^102^) and/or specific surface area measurements (e.g., via the BET method^103^).

## Translocation

### Bioturbation

Incorporation of organic matter from litter/organic horizons into the mineral soil, and its further relocation therein, is substantially affected by the bioturbation activity of soil fauna (broadly defined as “the enhanced dispersal of particles resulting from sediment [soil] reworking by burrowing animals”^10,104^). Soil fauna has been estimated to remove up to 73 and 100% of the annual litterfall from the soil surface in tropical and temperate ecosystems, respectively, which highlights the high relevance of bioturbation across biomes^32^. This transfer of organic matter to the mineral soil as plant fragments or feces (see “Transformation” section above) fuels soil food webs, biochemical processes, and likely plays an important role in the formation and dynamics of POM and MAOM (Fig. 2).

#### Earthworm burrows/drilosphere

Earthworms have a disproportionately large influence on bioturbation^105^, as they not only dominate this process in the majority of ecosystems^10,106^, but also directly influence the share between POM and MAOM via bioturbation. When fragmenting and removing litter from the soil surface and incorporating it into the mineral soil, which can increase the amount of POM in soil^107^, anecic (deep burrowers) and endo-epigeic (soil-/litter-dwelling) earthworm species create vertical and horizontal burrows, respectively^108,109^. Burrows of endogeic and endo-epigeic species are mostly confined to the upper mineral soil and frequently refilled with soil, while those of anecic earthworms are relatively stable and can occur at large soil depths (up to 3 m)^110^. Although these burrows only occupy a minor volume of the whole soil^111^, they can be hotspots for biochemical and physical processes^112–114^. For example, burrow walls are compacted though exertion of axial and radial pressures and mucus deposition by the earthworm^115^, resulting in smaller pores and pore neck diameters^116^, in which small POM may be less accessible to microbial decomposers^117^. Burrow walls have also been reported to feature high microbial biomass, enzyme activities, and C mineralization rates^118–122^, being hotspots for C turnover and, perhaps, the concurrent formation of MAOM via the microbial pathway. This is supported by higher amounts of sugars detected in burrow walls^123^. Moreover, direct sorption of earthworm mucus to reactive mineral surfaces in burrow walls can increase MAOM^124^. However, Don et al.^125^ did not observe enhanced adsorption of C to minerals in burrow walls, and the higher C stocks of burrow walls in that study mineralized relatively quickly (in 3-5 years). The net effect of POM stabilization, MAOM formation, and microbial decomposition on C in burrow walls remains to be explored (Fig. 3).

#### Ants, termites, and dung beetles

While earthworms have clearly been the focus of a plethora of studies, other taxa may have considerable effects on organic matter dynamics via their bioturbation activity as well^10,126–128^, particularly under conditions less favorable for earthworms, such as in acid forests or dry ecosystems. However, we have not found any study on how bioturbation of soil fauna other than earthworms influences the formation of and share between POM and MAOM, although especially the characteristic mound- and gallery-building activity of ants and termites or bioturbation of dung beetles could have distinct effects on these fractions.

For example, the bioturbation and nest-building activity of ants and termites may induce particle sorting, with relatively higher and lower amounts of clay in nests and the underlying soil, respectively^129–133^ (but see also Whitford and Eldritch^134^). This process has been reported to soil depths well exceeding 1 m^135,136^ and may even reverse lessivation (the downward movement of clay particles)^133^. This may substantially decrease the capacity of soils below ant and termite nests to store organic matter as MAOM. In contrast, the transport of clay particles from deeper soil layers, which are likely far from C-saturation^137^, to the surface may increase this capacity in nest soil. Particle sorting and incorporation of organic matter into the soil has also been reported for dung beetles, specifically for tunnelers. This may increase the contents of clay, likely originating from deeper soil layers^138,139^, C, and nutrients in soil^140^, potentially boosting MAOM formation through co-location of organic matter and reactive mineral surfaces and alleviation of microbial nutrient limitations (also in combination with the effects of beetles on dissolved organic matter; see below).

Transport of forage to ant and termite nests and preferential feeding of dung beetles may also influence POM. For example, litter- and wood-feeding ants and termites, respectively, transport “recalcitrant” compounds to their nests^133,141,142^, potentially resulting in higher amounts of POM as compared to those in the surrounding bulk soil or in nests of ants and termites feeding on less recalcitrant substrates, such as honeydew or grasses^141^. Termites have also been shown to substantially alter wood decay rates, with potential consequences for POM (and MAOM) in the underlying mineral soil^143^. Likewise, dung beetles preferentially ingest small particles (8–50 µm in size) and leave large plant fragments widely unaffected^144^, perhaps increasing the transfer of POM to the mineral soil upon bioturbation.

While the effects of dung beetle bioturbation on the share between POM and MAOM are likely confined to the soil below dung pads (to ~10 cm depth), those of ant and termite bioturbation could be traced beyond the perimeter of nests (up to 0.5 ha)^133,145^ and were still perceivable 20 years after nest abandonment^142^. However, it is difficult to make general statements about the influence of ant/termite bioturbation on POM and MAOM, as this influence will likely strongly be modulated by the respective environmental conditions (e.g., climate or soil properties). Such conditions have been shown to influence factors important to the decomposition of POM and formation of MAOM in nests, such as microbial activity, pH, and temperature^132,146–151^.

#### Other soil fauna

Other groups of soil fauna, apart from earthworms, ants/termites, and beetles, perform bioturbation as well^10^. However, much less is known about the relevance of bioturbation by those groups. For example, woodlice, millipedes, and springtails can significantly increase litter decomposition across litter species^152–162^. Recent evidence also suggests that invertebrates (and vertebrates) contribute globally to deadwood decay^163^. Since animals are able to use only a fraction of the ingested litter for their own nutrition, most of this litter is converted into feces^164^. Additionally, woodlice and millipedes have been reported to feed preferentially on leaf lamina, leaving the leaf vein mostly untouched^164^, which may increase the accumulation of recalcitrant compounds and POM at the soil surface. Animal species that actively move vertically may mix some of this litter and POM with the mineral soil (either as litter fragments or feces). Such vertical movement through the soil to forage, lay eggs, hibernate, or estivate has been reported for insect larvae and adults, millipedes, and centipedes^164–170^. The rate of this mixing, however, is yet to be quantified in controlled experiments, and in situ using various biochemical tools (Fig. 3), and linked to the formation MAOM and accumulation (or decomposition) of POM.

### Dissolved compounds

Some faunal taxa can have substantial effects on fluxes of dissolved organic matter and thus affect the direct sorption pathway of MAOM formation. For example, potworms dwelling in organic horizons, such as the forest floor or peat deposits, have often been related to increased production and leaching of dissolved organic matter^31,171–175^, likely via stimulation of microbial activity and thus decomposition of organic matter^176^. Increased leaching of dissolved organic matter from litter and forest floors has also been reported in the presence of springtails^177,178^, likely induced by feces production, and for dung pats reworked by beetles^179^. Given that this dissolved organic matter encounters reactive mineral surfaces, such as when percolating through forest or rangeland soils, soil fauna may indirectly influence sorption (or desorption^180^) of dissolved compounds to mineral surfaces and thus MAOM formation.

Dissolved organic matter rich in nutrients, such as that released from organic horizons or dung pats in the presence of potworms or beetles, respectively^171,175,181^, has also been reported to increase total nitrogen and phosphorus and ammonium-nitrogen in mineral soil^181–184^, potentially alleviating microbial nutrient limitations and boosting the microbial pathway of MAOM formation. This notion is supported by studies reporting microbial biomass stimulation in the presence of potworms and dung beetles^185–187^ (but see also Liiri et al.^175^ and Menendez et al.^179^). However, microbial traits considered important to the microbial pathway of MAOM formation, such as microbial C use efficiency^25^, have not been investigated in tandem with potworms or beetles and hardly with springtails^188^ (Fig. 3).

While the potential effects of beetles on the formation of MAOM via increased leaching of dissolved organic matter is relevant mainly in direct vicinity to dung pats (to ~10 cm depth)^181^, those of potworms and springtails may be important on larger scales. Potworms are typically abundant in wet, acid forest soils of the boreal and tundra zones, where they contribute up to 20 and 50% to the total animal biomass, respectively, while springtails are present in high abundance in virtually all ecosystems worldwide, being the most abundant in tundra soils^3^. Notably, the transfer of dissolved organic matter to deeper soil horizons can be enhanced by animals creating long and continuous vertical burrows, such as anecic earthworms^189^. Such preferential flow paths enable the rapid migration of dissolved organic matter, by which fauna could extend the relevance of dissolved organic matter to MAOM formation via direct sorption to deeper soil layers.

### Transport of bacteria and fungi

Microorganisms are key players in the decomposition of fresh organic matter and POM as well as the formation of MAOM. The mobility of microorganisms is generally limited, and any vector promoting microbial dispersal may help the exploitation of resources previously out of reach^190^. This may be specifically relevant for bacteria, as their mobility is generally more strongly limited than that of fungi^191–193^. Vertical and horizontal transport of bacteria, for example, by nematodes^194–196^, may have importance in SOM dynamics if influencing emergent traits relevant to MAOM formation and/or accumulation (or decomposition) of POM, such as microbial diversity or C use efficiency^25,197^. Moreover, transport of fungal spores in the gut or on the body surface has been reported for a magnitude of invertebrate soil faunal species (i.e., snails, termites, ants, woodlice, mites, potworms, springtails, nematodes, millipedes, beetles, and earthworms)^198–201^. Such transport might have a recognizable effect on SOM dynamics in ant and termite nests^202,203^ or in disturbed sites, such as post-mining areas, where it might affect plant establishment in early successional stages^204^. However, generalizable statements on whether certain faunal taxa preferentially (or exclusively) transport certain microorganisms and whether such transport is indeed relevant to the formation of POM and MAOM remain unsupported by a lack of studies and data^198^ (Fig. 3).

## Grazing on microorganisms

Microorganisms are the main food source for various faunal taxa, with preference for either fungi or bacteria^40,114^. Microorganisms can also be accidentally consumed by faunal taxa that prefer litter or SOM as food sources. Such targeted and incidental grazing can substantially alter microbial biomass, community composition, and activity, and thus potentially the amount and proportion of POM and MAOM in soils (Fig. 2).

### Grazing on bacteria

Bacteria are the preferred food source for protists (but see Geisen^205^) and some nematode species. Protists appear to generally decrease bacterial abundance in bulk and rhizosphere soil^206–208^. In turn, bacterial abundance was found to either increase or decrease upon nematode grazing^209,210^, which may relate to nematode density in soil as well as altered microbial community structure and growth dynamics in response to grazing^211,212^. However, a common response to grazing by both protists and nematodes is an increase in bacterial activity^196,208,213,214^, specifically of gram-negative bacteria for nematodes (both bacterivorous and fungivorous species)^8,196,210,214–216^ and gram-positive bacteria for protists^217–219^. Shifts in the dominance of these bacterial groups upon grazing^41^ may alter utilization of rather labile (gram-negative) and more complex (gram-positive) organic matter^220,221^. Dominance of either bacterial group may also affect the turnover time of bacterial biomass in soil, which can be ~40% longer for gram-positive as compared to gram-negative bacteria^222^. Because adsorption of necromass from gram-negative bacteria to microbial necromass already present on reactive mineral surfaces can be higher than that of necromass from gram-positive bacteria^223^, grazing may ultimately alter the formation of MAOM via the microbial pathway. Changes in microbial community composition in response to protist and/or nematode grazing, specifically in rhizosphere soil, can also alter root growth/architecture^224^ and exudation rates^225^ (see also “microbial loop”^41,194^), perhaps affecting accumulation of POM (via increased structural root inputs) or the direct sorption pathway of MAOM formation (via exudates). Protists may also actively contribute to microbial necromass formation by using the cytoplasmic contents of bacteria for growth and releasing undigested cell walls and other recalcitrant materials as waste^226^, which may then interact with minerals and form MAOM^227^. Increased excretion of extracellular polymeric substances by bacteria in response to grazing by protists has also been reported to foster aggregate formation^228^, which could render POM within more persistent.

### Grazing on fungi

Woodlice, mites, millipedes, springtails, and fungivorous nematodes generally prefer fungi over bacteria as their main food source^40,229–233^. This can have consequences for mycelial growth, the outcomes of interspecific competition^232,234–236^, fungal biomass^153,237^, and microbial diversity and community composition^8,216^. Earthworms can also preferentially feed on certain fungal species^238^, but the influence of this on soil microbial communities remains to be elucidated.

These multiple effects by fungal grazers on the soil microbiome may have various consequences for the stability of SOM. For example, although woodlice hampered mycelial growth, fungal decomposition of organic matter increased due to prevention of exclusion of other fungi and increased fungal diversity^239,240^. This may promote the microbial pathway of MAOM formation (and decomposition of POM), as microbial diversity has been linked to microbial necromass in MAOM^197^ and microbial C-use efficiency^241^, which in turn is related to the formation of MAOM^24,242^. Likewise, fungivorous nematodes can decrease microbial alpha diversity and C-use efficiency but increase the biomass of gram-negative bacteria^8,216^, with potentially opposite effects on MAOM formation. Finally, density-dependent promotion or impairment of arbuscular mycorrhizal (AM) or ectomycorrhizal (EM) fungal growth^243^, specifically by springtails^244^, may influence the share between POM and MAOM based on the respective nutrient acquisition strategy of these fungi. Arbuscular mycorrhizal fungi are unable to directly extract nitrogen and phosphorus from organic tissues, so that they release C to stimulate the growth and activity of saprotrophic microorganisms to “mine” these nutrients for them^245^. This can boost the microbial pathway of MAOM formation^246^. In contrast, EM fungi have retained some of their capacity to directly degrade organic matter so that competition for nutrients may suppress free-living saprotrophs^247^ and the decomposition of POM and formation of MAOM^248^. Thus, soils dominated by EM fungi will likely harbor more POM and less MAOM, while soils dominated by AM fungi will likely harbor more MAOM^245,246^. However, soil fauna generally appears to prefer saprotrophic over mycorrhizal fungi^230,243,249^, which might suppress decomposition of organic matter and, perhaps, foster the accumulation of POM. Recent evidence also suggests that impairment or promotion of individual fungal species by springtails can affect aggregate formation^250^ and thus the stability of POM *and* MAOM within^50^.

## Interactions across soil faunal taxa and influence of climate and land use

Faunal taxa do not live isolated in soil but coexist with other taxa embedded in complex soil food webs^251^. Interactions among fauna on higher trophic levels can trickle down through the food web and affect multiple taxa on various lower trophic levels^252^ and vice versa. However, since experimental studies on soil food webs are difficult, many studies have investigated how individual taxa or groups of taxa interact to suppress or boost each other’s biomass/abundance and/or activity. Such amensalism or commensalism has been reported for interactions between microarthropods, such as mites or springtails, and nematodes and potworms, and between earthworms or ants and microarthropods^113,253–258^. For example, the presence of microarthropods can reduce the densities of nematodes and potworms through predation, while endogeic earthworms can suppress microarthropod densities through amensalism^113^. In contrast, anecic earthworms and ants appear to create habitat conditions, such as stable burrows, middens, or nest soil rich in nutrients and microorganisms, that enable microarthropods to thrive^113,257^. The activity of certain faunal taxa can also alter the effect other taxa have on ecosystems. In the presence of isopods, and their feces in particular, endogeic earthworms preferentially fed on these feces^259^, which are energetically less demanding to process, and formed lower amounts of aggregates^160^. Similarly, termites reduced their foraging rates in response to ant predation, with potential consequences for the amount of POM in termite nests^260^. Such interactions, however, are likely strongly influenced by environmental conditions such as soil fertility, organic matter inputs, and specifically, land use and climate change^261,262^.

While wetter and warmer sites will likely experience an increase^263–270^ and those affected by drought a decrease in soil faunal biomass^266,268,271–276^, climate change effects are not necessarily uniform across faunal taxa^277^. For example, increased warming or precipitation resulted in higher diversity of fungivorous mites^278^ or increased the relative abundance of fungivorous nematodes^279^, indicating changes in food-web structure beyond mere increases or decreases in biomass. These effects may further be amplified (or dampened) by land-use change. Specifically, land-use intensification appears to consistently reduce the biomass, abundance, richness, and diversity of soil fauna, apparently largely consistently across taxa^277,280–288^.

We here clarify that the potential effects of individual soil faunal taxa on POM and MAOM do not necessarily reflect their effects when being part of the complex food webs encountered in the multitude of soil types present on Earth or when being affected by climate and/or land-use change. Studies that aim to mechanistically explore the role of soil fauna in SOM dynamics and stability ideally take these dependencies into account and, apart from investigating individual taxa, examine interactions among taxa under variable environmental conditions, such as in different soil types, under different land uses and land-use intensities, or in simulated future climates.

## Research gaps and recommendations

We emphasize that soil fauna may have a strong influence on the formation of and share between labile (POM) and stabilized (MAOM) SOM via three major processes – transformation, translocation, and grazing – with potentially far-reaching, global implications for management focused on increasing or maintaining soil C storage, nutrient stores, or crop yields. However, studies on the relevance of soil fauna to SOM stability beyond earthworms are widely lacking (Fig. 1). This might be due to the long history of ecological experiments with earthworms, their large body size and simple handling in the field and in the lab, their high biomass and ecosystem effects, as well as their significant contribution to decomposition processes^289^. We thus highlight the necessity to quantitatively assess the extent to which the wide diversity of soil fauna contributes to the formation (or decomposition) of POM and MAOM via each of the three major processes identified in this review (Figs. 2 and 3), how interactions among faunal taxa or within whole faunal communities alter this contribution, and whether it remains stable under varying environmental conditions, such as related to land-use or climate change. To this end, we recommend the use of controlled, micro- or meso-scale laboratory experiments^290–292^ along with standardized fractionation schemes^293,294^, which allow for separation of soil into compartments with varying stability, such as POM and MAOM. Potential constraints related to representative results from experiments with smaller organisms (meso-/microfauna) may be overcome by adapting the experimental size, duration, and number of replicates, and designing collaborative experiments distributed among research institutes. Such laboratory approaches will allow mechanistic insights into individual and combined effects of soil fauna on SOM dynamics, which can then be upscaled to field settings to investigate the relevance of land use, climate change, soil depth, or other environmental factors in this nexus^295^. Bioturbation rates and flows of C among different SOM pools (e.g., from litter to POM and aggregate-occluded POM to MAOM, from litter to dissolved organic matter to MAOM, and/or from litter to feces to MAOM) can be traced and quantified via stable isotope labeling, such as with ^13^C, coupled with compound-specific isotope measurements^296,297^ and physical fractionation^19,20,298^. This is specifically relevant for feces, as these structures, boosting microbial growth and leaching of dissolved organic matter, could strongly affect the formation of MAOM both via the microbial and direct sorption pathways. Likewise, the contribution of soil faunal necromass to SOM dynamics remains largely unknown but could be quantified by tracing the fate of isotopically labeled carcasses^299^. Combined with DNA analyzes^250^ of gut microbiomes, feces, and the surrounding soil, such isotopic and physical fractionation techniques could further provide insights into the relevance of microbial community changes to SOM dynamics and stability induced by soil fauna via grazing on microorganisms, transfer of gut microbiota to soil/feces, and physical and chemical transformation of organic matter in feces. Use of X-ray diffraction^102^ and/or BET analysis^103^, specifically in studies on termites (but also earthworms), would enable detailed insights into how mineralogical changes caused by soil fauna affect MAOM formation. Faunal effects on the whole C budget of soil should be quantified via monitoring of fluxes of C to and from the soil with different animal communities^300^, i.e., by quantifying heterotrophic respiration, leaching of dissolved organic matter, and C in bulk soil and/or fractions. The inclusion of living plants in controlled experiments could further help disentangle the role of soil fauna-plant interactions in SOM dynamics^290^, such as related to the microbial loop^41^. Finally, assessment and manipulation of soil faunal diversity in studies related to POM and MAOM could provide valuable insights into the relevance of biodiversity conservation efforts for establishing soils, as the most biodiverse systems on Earth^301^, as C sinks.

We are convinced that such experiments and the related knowledge gain are indispensable in accounting for the relevance of soil fauna in SOM dynamics. Environmental alterations are changing the composition and functioning of soil communities and processes^302^, which are key to soil C dynamics and feedbacks to climate change. Soil fauna are subject to many of these environmental changes^277^ and play decisive roles in SOM dynamics by regulating POM and MAOM formation through organic matter transformation, translocation, and grazing on microbial communities. Addressing this major research frontier requires novel interdisciplinary approaches to inform Earth system models and manage multifunctional soils in sustainable ways. We encourage cross-disciplinary cooperation among soil zoologists and chemists, microbial ecologists, and other related disciplines, as we envision that the vast remaining research gaps are most efficiently tackled with joint forces.

## Data Availability

No data have been generated in the preparation of this review.
